# A Carotenoid-Deficient Mutant in *Pantoea* sp. YR343, a Bacteria Isolated from the Rhizosphere of *Populus deltoides*, Is Defective in Root Colonization

**DOI:** 10.3389/fmicb.2016.00491

**Published:** 2016-04-18

**Authors:** Amber N. Bible, Sarah J. Fletcher, Dale A. Pelletier, Christopher W. Schadt, Sara S. Jawdy, David J. Weston, Nancy L. Engle, Timothy Tschaplinski, Rachel Masyuko, Sneha Polisetti, Paul W. Bohn, Teresa A. Coutinho, Mitchel J. Doktycz, Jennifer L. Morrell-Falvey

**Affiliations:** ^1^Biosciences Division, Oak Ridge National LaboratoryOak Ridge, TN, USA; ^2^Department of Chemical and Biomolecular Engineering, University of Notre DameNotre Dame, IN, USA; ^3^Department of Microbiology and Plant Pathology, Forestry and Agricultural Biotechnology Institute, University of PretoriaPretoria, South Africa

**Keywords:** *Pantoea*, carotenoids, crtB, indole-3-acetic acid, poplar, rhizosphere, zeaxanthin

## Abstract

The complex interactions between plants and their microbiome can have a profound effect on the health and productivity of the plant host. A better understanding of the microbial mechanisms that promote plant health and stress tolerance will enable strategies for improving the productivity of economically important plants. *Pantoea* sp. YR343 is a motile, rod-shaped bacterium isolated from the roots of *Populus deltoides* that possesses the ability to solubilize phosphate and produce the phytohormone indole-3-acetic acid (IAA). *Pantoea* sp. YR343 readily colonizes plant roots and does not appear to be pathogenic when applied to the leaves or roots of selected plant hosts. To better understand the molecular mechanisms involved in plant association and rhizosphere survival by *Pantoea* sp. YR343, we constructed a mutant in which the *crtB* gene encoding phytoene synthase was deleted. Phytoene synthase is responsible for converting geranylgeranyl pyrophosphate to phytoene, an important precursor to the production of carotenoids. As predicted, the Δ*crtB* mutant is defective in carotenoid production, and shows increased sensitivity to oxidative stress. Moreover, we find that the Δ*crtB* mutant is impaired in biofilm formation and production of IAA. Finally we demonstrate that the Δ*crtB* mutant shows reduced colonization of plant roots. Taken together, these data suggest that carotenoids are important for plant association and/or rhizosphere survival in *Pantoea* sp. YR343.

## Introduction

The rhizosphere is the site of a complex network of plant–microbe and microbe–microbe interactions which ultimately influence plant health and productivity. Microbes can be considered pathogenic, neutral, or beneficial depending on the plant host with which they associate (Raaijmakers et al., 2009). Among these bacteria is a group of plant growth-promoting bacteria (PGPB) that can colonize within the plant, on leaf or root surfaces, or in the surrounding rhizosphere (Hayat et al., 2010; Barret et al., 2011; Beneduzi et al., 2012). These bacteria can promote plant growth via phytohormone production, nitrogen fixation, and/or enhancement of water and mineral uptake (Kloos et al., 2001; Perrig et al., 2007; Bashan and de-Bashan, 2010; del Amor and Cuadra-Crespo, 2011). For example, the ability of microbes to influence root development via biosynthesis of the auxin indole-3-acetic acid (IAA) is well documented and multiple pathways for microbial IAA production have been described (Spaepen et al., 2007). Although well-studied genera like *Azospirillum* and *Rhizobium* are included in the PGPB group, the mechanisms by which these bacteria interact with and influence plant growth are not yet fully understood.

Recently the bacterial community associated with mature *Populus deltoides* roots was analyzed and shown to be dominated by *Proteobacteria*, *Acidobacteria*, and *Verrucomicrobia* (Gottel et al., 2011; Shakya et al., 2013). Among the organisms isolated from the *Populus* rhizosphere was the γ-proteobacterium *Pantoea* sp. YR343. Different species within the *Pantoea* genera have been shown to be either beneficial or harmful in association with plants (Walterson and Stavrinides, 2015). For example, *P. stewartii* and *P. ananatis* are responsible for Stewart’s bacterial wilt disease on corn, leaf blotch disease in Sudangrass, and center rot in onion (Azad et al., 2000; Walcott et al., 2002). *P. rwandensis*, *P. rodasii*, *P. vagans*, and *P. eucalypti*, have been implicated as the causal agents of bacterial blight, leaf lesions, and dieback in eucalyptus (Coutinho et al., 2002; Brady et al., 2009, 2012). In contrast, *P. vagans* C9-1 is used as a biocontrol agent to protect against fire blight (Stockwell et al., 2010) and *P. agglomerans* can protect against plant pathogens like *Pseudomonas syringae* pv. *Syringae* (Braun-Kiewnick et al., 2000). Strains of *P. agglomerans* have also been shown to promote plant growth in wheat, rice, and cotton (Ruppel et al., 1992; Amellal et al., 1998; Egamberdiyeva and Höflich, 2001; Verma et al., 2001; Feng et al., 2006). It is thought that *P. agglomerans* promotes plant growth by enhancing root architecture, which increases the amount of minerals and water that can be taken up by the plant (Sergeeva et al., 2007). *P. dispersa* and *P. agglomerans* have been shown to produce IAA and evidence supports the hypothesis that the production of IAA by *P. agglomerans* is responsible for its plant growth-promoting abilities (Sergeeva et al., 2007; Kulkarni et al., 2013). Additional studies have shown that production of exopolysaccharides by *P. agglomerans* contributes to soil aggregation and moisture control, which also enhances plant growth (Amellal et al., 1998).

Plants produce reactive oxygen species as a by-product of their metabolism, and these molecules have been implicated in plant defense mechanisms (Apel and Hirt, 2004), stress responses (Gechev et al., 2006), and the establishment of symbiotic relationships (Gechev et al., 2006). Many soil-dwelling and plant-associated bacteria, including *Pantoea* spp., produce carotenoids, which are pigment molecules found in the cellular membrane (Dussault et al., 2008; Mohammadi et al., 2012). Carotenoids have been studied for their photoprotective and antioxidant activities in some photosynthetic and plant growth-promoting microbes, where carotenoids serve an essential role in protection against singlet oxygen species, protection from oxygen during nitrogen fixation, and in energy transfer during photosynthesis (Foote and Denny, 1968; Cogdell and Frank, 1987; Hartmann and Hurek, 1988; Britton, 1989; Krinsky, 1989, 1993; Hirayama et al., 1994; Edge et al., 1997; Cogdell et al., 2000; Fraser et al., 2001). In addition, carotenoids can modulate membrane fluidity and may play a role in the formation of membrane domains (Huang and Haug, 1974; Chamberlain et al., 1991; Gruszecki, 1999; Gruszecki and Strzalka, 2005; Landrum, 2009; Lopez and Kolter, 2010). Zeaxanthin (both mono- and diglucoside forms) was found to be the predominant carotenoid present in *Erwinia herbicola* and *P. stewartii* (Hundle et al., 1991; Mohammadi et al., 2012). The carotenoid biosynthesis pathway has been well characterized in *Pantoea* and consists of six enzymes: geranylgeranyl diphosphate (GGPP) synthase CrtE, phytoene synthase CrtB, phytoene desaturase CrtI, lycopene cyclase CrtY, β-carotene hydroxylase CrtZ, and the zeaxanthin glucosyltransferase CrtX (To et al., 1994; Sedkova et al., 2005). In *P. ananatis*, it was found that deletion of the phytoene synthase gene, *crtB*, resulted in the loss of yellow pigment and increased sensitivity to environmental stress factors (Mohammadi et al., 2012).

Here we describe *Pantoea* sp. YR343, a non-pathogenic bacterial strain isolated from the rhizosphere of poplar, which was found to be a robust colonizer of plant roots. This motile, rod-shaped bacterium is able to solubilize phosphate and produce IAA. To better understand the molecular mechanisms involved in rhizosphere survival and plant association in *Pantoea* sp. YR343, we constructed a mutant in which the phytoene synthase gene, *crtB*, was deleted. As predicted, the *crtB* mutant is defective in carotenoid production and showed increased sensitivity to reactive oxygen species. In addition, we find that the Δ*crtB* mutant is impaired in other behaviors, including biofilm formation, production of IAA, and root colonization.

## Materials and Methods

### Bacterial Isolation and Growth Conditions

Fine roots and associated rhizophere samples were harvested from native *Populus deltoides* trees at the Yadkin River in North Carolina in May 2011 and bacteria were isolated from both the endosphere and rhizosphere. A rhizosphere isolate designated YR343 was restreaked three times to R2A agar plates (R2A Agar, VWR) to obtain a purified strain and a draft genome sequence was obtained (Brown et al., 2012). This bacterial strain is referred to as *Pantoea* sp. YR343 in this paper. *Pantoea* sp. YR343 was cultured at 28°C in R2A medium (R2A Broth Premix, TEKnova, Inc.) or on R2A agar plates. Cultures were also grown in standard LB, M9, TY (per 1 l, 10 g tryptone, 5 g yeast extract), or SOBG medium (per 1 l, 20 g tryptone, 5 g yeast extract, 0.5 g NaCl, 2.4 g MgSO_4_, 0.186 g KCl, 50 ml of 40% v/v glycerol). Fluorescent strains of *Pantoea* sp. YR343 expressing EGFP were engineered either by expressing EGFP from a Gateway-modified pBBR1-MCS5 plasmid (Pelletier et al., 2008) maintained with 10 μg gentamycin ml^-1^ (referred to as YR343-pGFP) or by chromosomal insertion of EGFP using pBT270 (pUC18-miniTn7T2-PA1/04/03-GFP, a gift from B. S. Tseng, University of Washington) (referred to as YR343-GFP).

### Phylogenetic Analysis

Approximately 500 base pairs from the 5′ end of the 16S rRNA gene were amplified using universal primers (Weston et al., 2012). Initial BLAST (16S and gyrB) and Ribosomal Database Project 16S sequence comparisons suggested a strong affiliation of strainYR343 with *Pantoea* spp. Multi-locus sequence analysis (MLSA) of partial nucleotide sequences of genes *rpoB*, *infB*, *atpD* and *gyrB* was employed as described (Brady et al., 2008, 2010, 2012). Sequences from *Populus* rhizosphere isolates GM01, YR343 (Brown et al., 2012) and three other unidentified isolates for which data was available from genome sequencing efforts on IMG were aligned using the Translation alignment tool within Geneious^TM^ (v6.0.4-www.geneious.com) with 94 total representatives of *Pantoea* and related taxa of *Erwinia*, *Tatumella* and other *Enterobacteriaceae* for further phylogenetic analysis with *Chronobacter sakazakii* as an outgroup. Maximum likelihood analysis was conducted using PhyML v3.0, (Guindon et al., 2010) as implemented within Geneious^TM^. A general time reversible model was used with each rate category and allowed to vary according to a gamma distribution. An initial best tree was obtained via comparison of a neighbor-joining tree using NNI/SPR analysis, and 100 bootstrap resampling trees were conducted.

### Pathogenicity Assay with *Pantoea* sp. YR343 in *Populus deltoides*

*Populus deltoides* WV94 plants were grown from shoot tips that were surface-sterilized by washing for 5 min in a 1% Tween-20 solution, followed by a 1 min wash in 70% ethanol, then a 12 min wash in 10% bleach. Shoot tips were then washed three times in sterile water before inoculation into MS medium (per 1 l of medium: 4.43 g MS salts, 0.5 g MES hydrate, 30 g sucrose, 5 g activated charcoal, 1.5 g Gelrite and 1 ml plant preservative mixture (PPM). The pH was adjusted to 5.7 using KOH prior to addition of activated charcoal and Gelrite). Once the shoot tips had rooted, the Poplar plants were transferred to Magenta boxes containing sterile clay soil mixed with 1X Hoagland’s solution (1.63 g Hoagland Modified Basal Salt Mixture per 1 l water, Phytotechnology Laboratories) to provide plant nutrition. For this assay, there were two treatments using three plants per experiment, along with a set of three control plants. In the first experiment, the clay soil was mixed with approximately 1 × 10^9^ cells of *Pantoea* sp. YR343 grown in R2A medium prior to planting the newly rooted Poplar plant. For the second experiment, we used a culture with 1 × 10^8^ cells per ml of *Pantoea* sp. YR343. The control plants were treated by mixing R2A medium in the clay soil (at a similar volume to those treated with *Pantoea*) and also with R2A medium swabbed onto leaves. Plants were grown for 21 days at 24°C with a 12 h light and 12 h dark photoperiod. All plant samples were measured at the beginning of the study and again at the time of harvest in order to assess the effects of *Pantoea* sp. YR343 on such characteristics as stem height, leaf number and size, and root area.

### Phenotypic Analysis

To compare growth rates of *Pantoea* sp. YR343 in LB, R2A, or M9 media, overnight cultures were diluted 1:100 into fresh medium and 200 μl was loaded into 12 wells on a honeycomb plate and placed into a Bioscreen C Reader System at room temperature with shaking overnight. Phosphate solubilization was examined using Pikovskaya’s agar medium plates (per 1 l, 0.5 g yeast extract, 10 g dextrose, 5 g Ca_3_(PO_4_)_2_, 0.5 g (NH_4_)_2_SO_4_, 0.2 g KCl, 0.1 g MgSO_4_⋅7H_2_O, 0.0001 MnSO_4_⋅H_2_O, FeSO_4_⋅7H_2_O, 15 g agar) and incubated at 28°C for approximately 1 week prior to imaging. The presence of cellulose in *Pantoea* sp. YR343 exopolysaccharides was detected by inoculating 200 μl of an overnight culture into a glass-bottom dish containing 5 ml of R2A and grown statically at 25°C for 72 h. After incubation, the culture was stained with a solution of Calcofluor White (5 μg ml^-1^) (Sigma–Aldrich), and 5 μM SYTO61 (Life Technologies). Cultures were imaged using a Zeiss LSM 710 laser scanning confocal microscope and images were processed using Zen software (Zeiss). Swimming and swarming motility was examined on LB containing 0.3% w/v agar or 0.6% w/v agar supplemented with 0.4% w/v glucose or 0.4% v/v glycerol, based on previous studies (Herrera et al., 2008).

### Root Colonization Assays

*Arabidopsis thaliana* ecotype Col-0 seeds were germinated and grown, as previously described (Wang et al., 2005). Seeds were surface-sterilized by washing in a 15% bleach solution containing 0.01% Tween-20 for 15–20 min, then rinsed in sterile water. Afterward, seeds were washed briefly in 70% ethanol and rinsed several times in sterile water. Sterilized seeds were soaked in double distilled H_2_O containing 0.1% w/v agar at 4°C for 4 days and then germinated on Murashige and Skoog (MS) agar containing 0.25% w/v sucrose (Murashige and Skoog, 1962). Seedlings were incubated in a growth chamber at 24°C with a 12 h light and 12 h dark photoperiod. After 7 days, 4 – 6 *A. thaliana* seedlings of equivalent root lengths were transferred to new MS agar plates containing 0.25% w/v sucrose and grown for an additional 7 days. An overnight culture of *Pantoea* sp. YR343 was then pipetted in a line across the MS plate approximately 1 cm from the bottom edge, while sterile R2A media was used for the untreated control. After 10 days, plants were imaged for differences in root architecture. For analysis of root colonization, we utilized the YR343-pGFP strain. Co-cultured seedlings were harvested and mounted on slides prior to imaging with a Zeiss LSM710 confocal laser scanning microscope.

*Populus deltoides* WV94 shoot tips were surface-sterilized and grown as described for the pathogenicity studies. Rooted cuttings were planted in sterile clay soil inoculated with *Pantoea* sp. YR343-pGFP at 2.7 × 10^7^ CFU g^-1^ soil and incubated at 24°C with a 12 h light and 12 h dark photoperiod for 7 days. Roots and soil were collected for re-isolation of *Panteoa* sp. YR343 and measurement of colonization. Plants that were inoculated with medium alone were used as controls for bacterial contamination, which was not observed in these assays. Briefly, roots were weighed, washed by vortexing at least 30 s with 3 ml of PBS and a few small glass beads and dilutions of the wash solution was plated on R2A to measure colony forming units (CFU). For soil samples, 1 g of soil was mixed with 4 ml of PBS, then vortexed and plated similarly.

*Triticum aestivum* (wheat) seeds were sterilized as described for *Arabidopsis thaliana*, then incubated on wet filter paper in the dark for 3 days. Overnight cultures of wild type *Pantoea* sp. YR343-GFP and the Δ*crtB* mutant were normalized to the same optical density (OD_600_). Cultures were then inoculated into sterile, molten Fahraeus medium (per 1 l, 100 mg CaCl_2_⋅H_2_O, 120 mg MgSO_4_⋅7H_2_O, 100 mg KH_2_PO_4_, 150 mg Na_2_HPO_4_, 5 mg Ferric citrate, and a trace amount of Na_2_MoO_4_⋅2H_2_O, then adjusted to pH 7.5 and added 4 g agar) that was cooled, but not solidified, and then the medium was poured into sterile glass test tubes or magenta boxes and allowed to solidify. Plants treated with sterile R2A medium were used as controls for background contamination. Afterward, germinated seedlings were added to each tube. Three plants were measured for colonization per treatment: uninoculated control, wild type only, Δ*crtB* only, and a 1:1 mix of wild type and Δ*crtB*. Each plant was inoculated with approximately 1 × 10^8^ cells per plant and grown for 1 week prior to harvesting. Roots were harvested as described above for *Populus* and analyzed for CFUs per gram of root material. Mixtures of wild type and the Δ*crtB* mutant were distinguished by colony color (wild type colonies were yellow, while the mutant colonies were white). Imaging of root colonization was performed by staining root tissue with 5 μM Syto61 (Life Technologies) to stain all bacterial cells that were attached to the root. Upon rinsing, the roots were visualized with a Zeiss LSM710 confocal laser scanning microscope. Wild type cells were distinguished by fluorescence in both the red (Syto61) and green (EGFP) channels, whereas the Δ*crtB* mutant fluoresced only in the red channel (Styo61).

### Construction of *crtB* Mutant

A marker-less mutant lacking the phytoene synthetase gene, CrtB (PMI39_03408), was generated by PCR amplification of the DNA regions 500 base pairs upstream (using primers CrtB_up For (*Xba*I): 5′-GCTCTAGATCCGCGTCCACCTTT-3′ and CrtB up Rev (*Xho*I): 5′-CCGCTCGAGTCTTACGTCCGTGGC-3′) and 500 base pairs downstream (using primers CrtB_dn For (*Xho*I): 5′-CCGCTCGAGTCGGCGCGATCCTCC-3′ and CrtB_dn Rev (*Xba*I): 5′-GCTCTAGATGTTTCGGTCCGCGC-3′) of the *crtB* gene from *Pantoea* sp. YR343. PCR products were ligated into pK18mobsacB (Schafer et al., 1994) and the resulting plasmid was verified by restriction digests and transformed into *Pantoea* sp. YR343 by electroporation and selected on R2A plates containing 50 μg kanamycin ml^-1^. Colonies lacking yellow pigmentation were screened by PCR to verify that the *crtB* gene was deleted.

### Carotenoid Extraction and Analysis

Carotenoids were extracted from the wild type *Pantoea* sp. YR343 and Δ*crtB* mutant as described (Mohammadi et al., 2012). Briefly, 2 ml of cells grown in LB medium were rinsed in water, resuspended in 1 ml of 100% methanol, and heated at 85°C for 20 min. After centrifugation to remove the cell debris, spectral analysis of the extracted carotenoids was measured in triplicate in the range of 400–500 nm using a BioTek Synergy 2 microplate reader.

### Cell Viability Assays

Wild type *Pantoea* sp. YR343 and Δ*crtB* were grown in LB medium at 28°C overnight. Cells were diluted 1:20 into fresh LB and grown 2–3 h at 28°C with shaking. In a 96 well plate, 100 μl of log phase or stationary phase cells were treated with final concentrations of 0.5, 1, 2, 5, 10, and 100 mM hydrogen peroxide and incubated for 1 h with shaking at 28°C. Afterward, each well was treated with 100 μl of Bac-Titer Glo reagent (Promega) according to the manufacturer’s instructions and luminescence was measured on a BioTek Synergy 2 microplate reader.

### Raman Spectroscopy

Single bacterial colonies grown on R2A agar plates were transferred to 2 ml of sterile PBS, vortexed, and adjusted to OD_600_ = 0.2 using a Cary UV-Vis spectrophotometer. 10 μl of the cell suspension was pipetted onto a clean gold-coated silicon wafer and dried for Raman analysis. Raman measurements were performed at room temperature using a confocal Raman microscope (Alpha 300R, WITec GmbH, Germany) equipped with a focused Nd:YAG operating at (aaa = 532 nm), a 40×, Nikon air objective (NA = 0.6), and a coverslip-corrected Nikon water immersion 60× objective (NA = 1). The laser radiation was delivered via a single mode optical fiber through a dichroic beam splitter into the microscope objective and focused to a diffraction limited spot size on the surface of the sample. The scattered Raman radiation was collected by the same objective and focused into a 50 μm diameter multi-mode fiber connected to a UHTS 300 spectrometer equipped with a 600 groove mm^-1^ grating and a back-illuminated CCD camera (Newton DU970 N-BV, Andor Inc., cooled to -65°C). Each Raman spectrum recorded was an accumulation of 100 spectra acquired with integration time of 0.5 s each at 5 mW incident laser power. For each sample, individual Raman spectra were collected at 15 randomly selected spatial locations and averaged to give the representative spectrum for that sample. Data analysis was performed using commercial graphic software Igor Pro 6.32A (Lake Oswego, OR, USA).

### Indole-3-Acetic Acid (IAA) Production Assay

Indole-3-acetic acid production was measured by a colorimetric assay as previously described (Tang and Bonner, 1947). Briefly, 500 μl of overnight cultures was diluted into 50 ml of M9 minimal medium plus L-Tryptophan (200 μg ml^-1^ final concentration), and incubated overnight at 28°C. Cells were harvested at the same OD and IAA was detected in the supernatant using Salkowski’s reagent (500 ml dH_2_O, 300 ml concentrated H_2_SO_4_, 2.03g FeCl_3_⋅6H_2_O) and absorbance was measured at 535 nm using a BioTekSynergy 2 microplate reader. All measurements were performed in triplicate and compared to a standard curve generated from IAA (Sigma–Aldrich). Gas chromatography-mass spectrometry was performed to confirm the presence of IAA using supernatants from wild type and mutant cultures grown in M9 minimal medium with or without L-Tryptophan as described (Weston et al., 2012).

### Biofilm Formation Assay

Biofilm formation was measured using the protocol described (O’Toole and Kolter, 1998), with the following modifications. An overnight culture was diluted 1:100 into either LB, R2A, or M9 medium supplemented with 0.4% w/v glucose and grown statically in a 96-well plate covered with breatheable tape in place of the lid (Breathe-EASIER, Diversified Biotech) at 28°C for 72 h. After 72 h, adherent cells were stained with 0.1% w/v crystal violet stain, then the crystal violet associated with biofilms was dissolved using a modified solution which contained 10% w/v SDS dissolved in 80% v/v ethanol (Tram et al., 2013). Absorbance was measured at 550 nm using a BioTekSynergy 2 microplate reader.

## Results

### Phylogenetic Analysis of *Pantoea* sp. YR343

**FIGURE 1:**
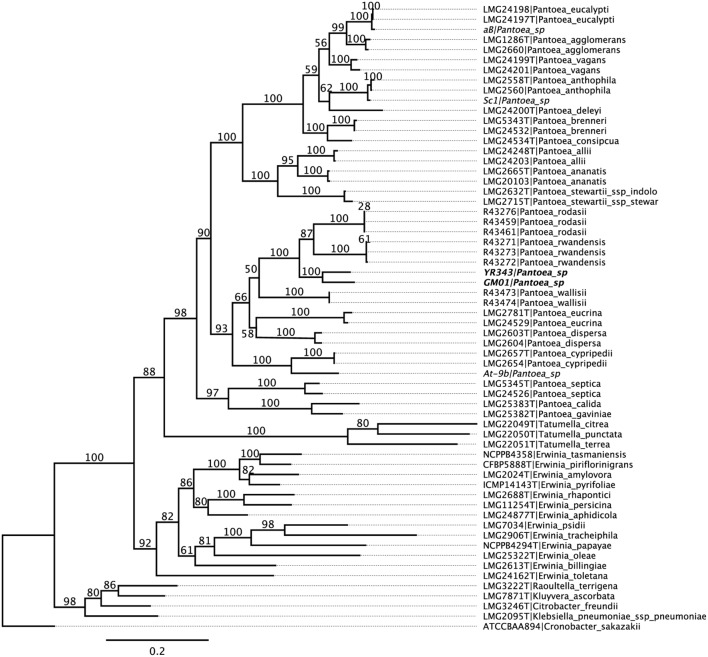
**Phylogenetic tree of *Pantoea* sp. YR343 and related species within *Enterobacteriaceae*.** Partial nucleotide sequences from *atpD*, *infB*, *gyrB*, and *rpoB* were used for multi locus sequence analysis.

*Pantoea* sp. YR343 was isolated from the rhizosphere of a native *Populus deltoides* tree growing in the Yadkin River region of North Carolina (Shakya et al., 2013) and a draft genome sequence was obtained (Brown et al., 2012). Phylogenetic analysis was conducted to compare the *Pantoea* isolate with known species within the *Enterobacteriaceae*. The results of multi locus sequence analysis (MLSA) using partial nucleotide sequences from *atpD*, *infB*, *gyrB*, and *rpoB* (Brady et al., 2012) revealed that *Pantoea* sp. YR343 clusters (100% of bootstrap replicates) with another *Pantoea* strain isolated from the Poplar rhizosphere (*Pantoea* sp. GM01; Brown et al., 2012) within a monophyletic *Pantoea* group (90%) and forms a unique, well supported (100%) group, basal to the recently described *P. rwandensis* and *P. rodasii*, that are known to form lesions on leaves of plantation-grown *Eucalyptus* trees (Brady et al., 2012) (**Figure 1**).

Although *Pantoea* sp. YR343 was isolated from a healthy poplar tree, its close phylogenetic relationship to *Pantoea* strains found to be the causal agents of leaf blight in *Eucalyptus* prompted us to test the effect of *Pantoea* sp. YR343 on cultured poplar cuttings. *Populus deltoides* WV94 cuttings were grown under sterile conditions in magenta boxes and exposed to *Pantoea* sp. YR343 at the roots by inoculation into the soil, or on leaves by direct foliar application. No evidence of lesions was observed on the leaves for 21 days following exposure to *Pantoea* sp. YR343 (data not shown). After 3 weeks, plants were harvested and measured for number of leaves, total leaf area (cm^2^), stem height (cm), and root area (cm^2^) (**Table 1**). Statistical analysis using a *t*-test indicated no significant differences (*p*-values >0.05) between control plants, foliar treated plants, or soil inoculated plants, suggesting that *Pantoea* sp. YR343 is not pathogenic to *P. deltoides* WV94.

**Table 1:** Effect of wild type *Pantoea* sp. YR343 on *Populus deltoides* WV94 growth.

Sample treatment	Leaf number	Total leaf area (cm^2^)	Stem height (cm)	Root area (cm^2^)
Uninoculated	2.67 ± 1.15	12.45 ± 12.45	2.99 ± 1.39	14.35 ± 7.58
Soil^a^	2.67 ± 0.58	13.30 ± 2.69	1.58 ± 0.44	10.31 ± 3.84
Leaf^b^	3.33 ± 1.53	16.98 ± 3.44	2.04 ± 0.32	10.44 ± 1.51

*Values represent the average differences in samples before and after treatment. ^a^Populus plants were inoculated by mixing bacterial culture with the soil prior to addition of the plant. ^b^Populus plants were inoculated by swabbing bacterial culture on the leaves of individual plants and then planting into sterile soil.*

### Phenotypic Properties of *Pantoea* sp. YR343

**FIGURE 2:**
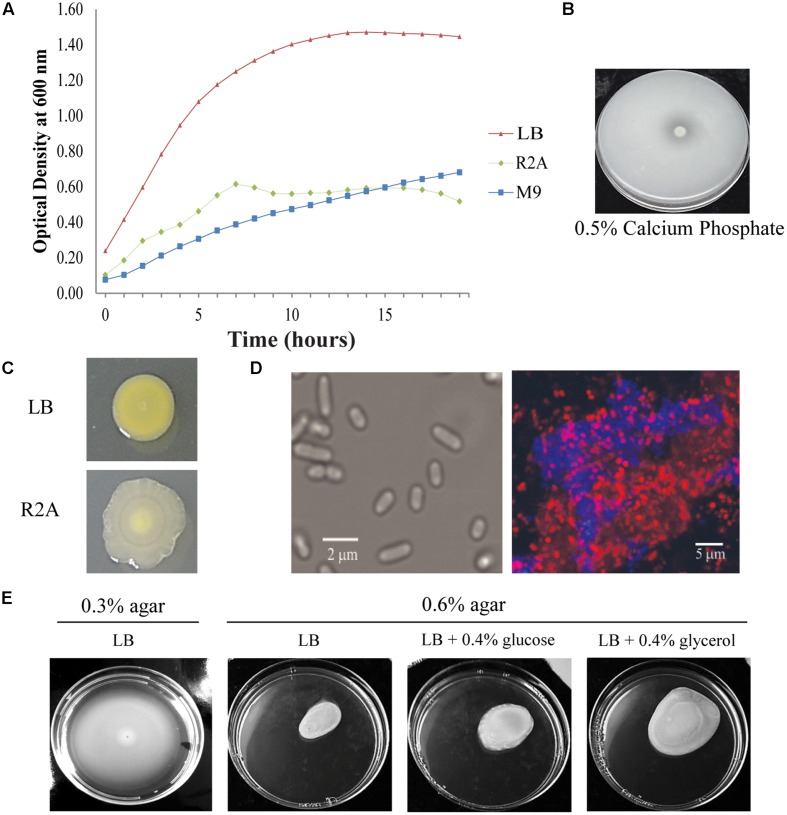
**Phenotypic characteristics of *Pantoea* sp. YR343. (A)** Growth of wild type *Pantoea* sp. YR343 grown in liquid LB, R2A, and M9 media. **(B)** Phosphate solubilization by *Pantoea* sp. YR343 is shown by a zone of clearing surrounding the colony. **(C)** Colony morphology of *Pantoea* sp. YR343 on LB (top) versus R2A (bottom) agar plates. **(D)** Bright field image of *Pantoea* sp. YR343 grown in R2A medium (left). (Right) Cellulose production in *Pantoea* sp. YR343 biofilm as detected by Calcofluor White staining (blue). The distribution of cells is indicated by staining with Syto61 (red). **(E)** Swimming and swarming motility of *Pantoea* sp. YR343. Swimming motility was characterized using LB medium with 0.3% agar (left) and swarming motility was characterized on LB medium with 0.6% agar, or on LB medium with 0.6% agar supplemented with 0.4% glucose or glycerol (right).

As determined by phylogenetic analysis, *Pantoea* sp. YR343 is a member of the *Enterobacteriaceae* family and falls into the class of γ-proteobacteria. *Pantoea* sp. YR343 is a Gram-negative bacterium that grows in liquid LB, R2A, or M9 minimal medium using glucose as a carbon source (**Figure 2A**). When grown on medium containing an insoluble form of calcium phosphate, a zone of clearing formed around colonies of *Pantoea* sp. YR343, indicating that this species is capable of phosphate solubilization (**Figure 2B**). *Pantoea* sp. YR343 growing on LB agar plates produced colonies that were round, smooth, and produced a yellow pigment (**Figure 2C**). In contrast, *Pantoea* sp. YR343 produced colonies that were irregularly shaped, wrinkly, and light yellow in color when grown on R2A agar plates (**Figure 2C**). *Pantoea* sp. YR343 also appeared to be highly mucoid, particularly when grown on R2A medium. Microscopic observation showed that *Pantoea* sp. YR343 displays a rod-shaped morphology with cells averaging approximately 2 μm in length (**Figure 2D**). In addition, *Pantoea* sp. YR343 was able to form biofilms on abiotic surfaces and the production of cellulose was detected in these biofilms by staining with Calcofluor White (**Figure 2D**). We also analyzed motility behavior in this organism using LB medium containing either 0.3% (swimming) or 0.6% (swarming) agar (**Figure 2E**). After 18 h, the cells had moved from the center to the edges of the plate, consistent with swimming motility behavior. Swarming motility was also observed, particularly when *Pantoea* sp. YR343 was grown on LB supplemented with 0.4% glycerol (**Figure 2E**). Finally, we used GC-MS analyses to examine whether *Pantoea* sp. YR343 could produce IAA, which is synthesized from the amino acid tryptophan (Patten and Glick, 1996). The tryptophan-dependent production of IAA was confirmed by GC-MS analyses and measured at approximately 0.5 μg/ml.

### *Pantoea* sp. YR343 Colonizes Plant Roots

**FIGURE 3:**
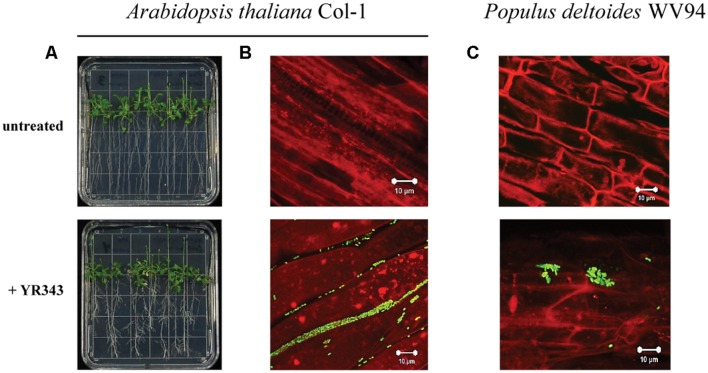
**Colonization of plant roots by *Pantoea* sp. YR343. (A)** *Arabidopsis thaliana* plants were grown on MS plates in the absence or presence of *Pantoea* sp. YR343 for 10 days prior to imaging. **(B)** Corresponding to the pictures in **(A)** are images of roots taken with confocal microscopy. The image on top shows roots from an uninoculated plant, while the bottom image shows roots colonized by YR343-pGFP. **(C)** *Populus deltoides* WV94 cuttings were grown in the presence or absence of *Panteoa* sp. YR343 expressing GFP. (Top) untreated *P. deltoides* WV94 plant; (bottom) *P. deltoides* WV94 cultured with YR343-pGFP for 7 days. In all images, *Pantoea* sp. YR343 is detected by GFP fluorescence (green) and plant roots are detected using autofluorescence (red).

We next analyzed the ability of *Pantoea* sp. YR343 to colonize plant surfaces. Co-cultures of *Arabidopsis* seedlings and *Pantoea* sp. YR343 showed that seedlings grown in the presence of the microbe had enhanced lateral root density compared to control seedlings (**Figure 3A**). To enable detection of *Panteoa* sp. YR343 on plant roots, we constructed a fluorescent strain (YR343-pGFP) in which GFP was expressed from a Gateway-modified pBBR1 plasmid (Pelletier et al., 2008). Using this fluorescent strain, we found that YR343-pGFP readily attached to the surface of *Arabidopsis* roots (**Figure 3B**). The same fluorescent strain was then used to determine whether *Pantoea* associated directly with *Populus* roots. Sterile *P. deltoides* WV94 cuttings were exposed to YR343-pGFP at the roots by inoculation into the soil at a concentration of 2.7 × 10^7^ CFU g^-1^ of soil. YR343-pGFP was re-isolated from the roots and soil at day 7 to determine its distribution and confirm viability on plants and in the soil. *Pantoea* sp. YR343 was preferentially associated with the plant as indicated by 3.5 × 10^6^ CFU g^-1^ of root, compared to 3.0 × 10^2^ CFU g^-1^ of soil. Consistent with this data, we observed YR343-pGFP cells attached to *P. deltoides* roots using confocal microscopy, whereas no bacteria were detected in the un-inoculated control plants (**Figure 3C**). Indeed, numerous locations along the roots were covered in bacteria. The bacteria were aggregated into what appeared to be biofilms on the surface of the roots and along the root hairs. Because the roots underwent several washes when they were harvested, the bacteria colonizing the *Populus* roots were firmly attached, as the transiently attached cells were presumably washed away.

### *Pantoea* sp. YR343 Produces Zeaxanthin Which Plays a Role in Withstanding Oxidative Stress

**FIGURE 4:**
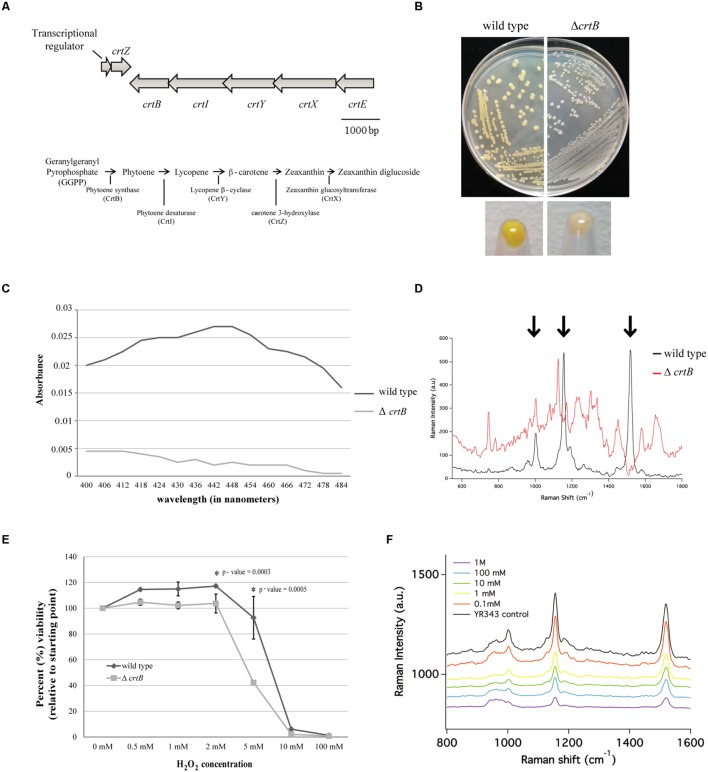
**Identification and characterization of a carotenoid mutant in *Pantoea* sp. YR343. (A)** Top, genomic structure of operon regulating carotenoid production. Bottom, predicted carotenoid biosynthesis pathway in *Pantoea* sp. YR343 based on genomic comparisons. PMI39_03412 encodes a product with 60% amino acid identity and 74% amino acid similarity to *P. ananatis* CrtE; PMI39_03408 encodes a product with 66% amino acid identity and 76% amino acid similarity to *P. ananatis* CrtB; PMI39_03409 encodes a product with 81% amino acid identity and 87% amino acid similarity to *P. ananatis* CrtI; PMI39_03410 encodes a product with 58% amino acid identity and 74% amino acid similarity to *P. ananatis* CrtY; PMI39_0340 encodes a product with 84% amino acid identity and 92% amino acid similarity to *P. ananatis* CrtZ, and PMI39_03411 encodes a product with 53% amino acid identity and 61% amino acid similarity to *P. ananatis* CrtX. **(B)** LB plates streaked with wild type *Pantoea* sp. YR343 (left) and Δ*crtB* (right) and grown for 48 h showing loss of pigmentation in the mutant strain. **(C)** Methanol extraction of carotenoids from wild type *Pantoea* sp. YR343 and Δ*crtB*. Graph represents the range of absorbances between 400 and 500 nm measured from one of two replicates. **(D)** Raman spectroscopy of wild type *Pantoea* sp. YR343 and Δ*crtB*. The wild type strain shows a spectra dominated by peaks (highlighted by arrows) corresponding to zeaxanthin. These peaks are reduced in the Δ*crtB* mutant. **(E)** Sensitivity of wild type and Δ*crtB* mutant cells to increasing concentrations of hydrogen peroxide. Cell viability was measured using the Bac-Titer Glo assay and plotted as a percentage relative to the untreated control, measured as 100%. **(F)** Raman spectroscopy of wild type *Pantoea* sp. YR343 treated with different concentrations of hydrogen peroxide shows a decrease in peak intensity at 1500 cm^-1^ upon treatment with hydrogen peroxide.

*Pantoea* sp. YR343 produced a yellow pigment under all growth conditions tested; however, it was the most apparent when cells were grown to stationary phase in LB medium. Genomic comparisons of the carotenoid biosynthesis operon in *Pantoea* sp. YR343 and *P. ananatis* LMG 20103 (De Maayer et al., 2010) indicated that the amino acid sequences of each gene from *Pantoea* sp. YR343 was more than 50% identical to those from *P. ananatis* LMG20103. Moreover, the carotenoid biosynthesis genes in *Pantoea* sp. YR343 were also arranged with an operon structure similar to that of *P. ananatis* and *P. stewartii* (Sedkova et al., 2005) (**Figure 4A**).

Since carotenoids play a role in resistance to reactive oxygen species and, thus, may influence plant association, we constructed a mutant defective in carotenoid production by deleting the *crtB* gene that encodes for phytoene synthase, the enzyme responsible for the conversion of geranylgeranyl pyrophosphate into phytoene which serves as a precursor for the synthesis of carotenoids (Sandmann and Misawa, 1992). As predicted, the Δ*crtB* mutant no longer produced a yellow pigment and the colonies appeared white (**Figure 4B**). This was further confirmed by analyzing the spectroscopic profiles of pigments extracted from wild type and Δ*crtB* mutant cells. The pigments extracted from wild type cells showed a spectra consistent with zeaxanthin as has been described for other *Pantoea* strains (Hundle et al., 1991; Mohammadi et al., 2012). Conversely, little to no pigment was extracted from the Δ*crtB* mutant (**Figure 4C**). We chose to further characterize this carotenoid using Raman spectroscopy. A representative Raman spectrum of wild type *Pantoea* sp. YR343 and the Δ*crtB* mutant from 550 cm^-1^ to 1800 cm^-1^ is shown in **Figure 4D**. The spectrum for the wild type exhibits three prominent bands at 1520 cm^-1^, 1155 cm^-1^, and 1002 cm^-1^ which are characteristic of carotenoid compounds, and arise from in-phase C = C and C–C stretching and in-plane CH_3_ rocking vibrations, designated ν_1_ = 1520 cm^-1^, ν_2_ = 1155 cm^-1^, and ν_3_ = 1002 cm^-1^ (Merlin, 1985; Withnall et al., 2003; Schulz et al., 2005; Goodwin et al., 2006; de Oliveira et al., 2010). In contrast, these bands are less prominent in the Δ*crtB* mutant.

We then tested the protective role of this carotenoid under oxidative stress created by exposure to hydrogen peroxide. Compared to the wild type strain, the Δ*crtB* mutant was more sensitive to the effects of increasing concentrations of hydrogen peroxide as determined by a viability assay (**Figure 4E**). To demonstrate that hydrogen peroxide has a specific effect on carotenoids, we examined the effect of hydrogen peroxide on wild type cells using Raman spectroscopy. By this method, we observed a steady decrease in the spectral peak intensity corresponding to zeaxanthin (**Figure 4F**) which correlates to the loss of cell viability. Furthermore, in contrast to the viability assays which show a precipitous decrease in viability only at higher concentrations of 5–10 mM H_2_O_2_, the Raman spectra in **Figure 4F** clearly show progressive loss of carotenoid-related peaks beginning at H_2_O_2_ concentrations as low as 1 mM.

#### The *crtB* Mutant is Defective in Biofilm Formation, Indole-3-Acetic Acid Production, and Plant Colonization

**FIGURE 5:**
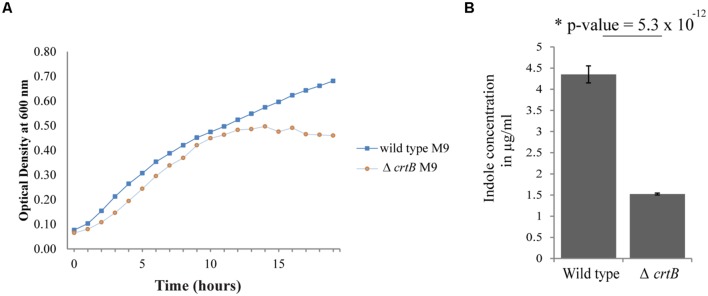
**Loss of carotenoids affects growth and phytohormone production. (A)** Growth curves of wild type *Pantoea* sp. YR343 and Δ*crtB* in M9 minimal medium. **(B)** Comparison of indole-3-acetic acid production in the wild type *Pantoea* sp. YR343 and the Δ*crtB* mutant. The production of indoles was measured using a colorimetric assay.

Since carotenoids have also been implicated in modulating membrane properties (Huang and Haug, 1974; Chamberlain et al., 1991; Gruszecki, 1999; Gruszecki and Strzalka, 2005; Landrum, 2009; Lopez and Kolter, 2010), we next examined whether this carotenoid affected other biological behaviors, such as growth, IAA production, biofilm formation, and root colonization. Comparisons of growth in liquid cultures between wild type and the Δ*crtB* mutant showed that the growth rates were very similar when cells were grown in LB or R2A medium (data not shown), but that the Δ*crtB* mutant reached stationary phase at a lower cell density compared to wild type cells when grown in M9 minimal medium (**Figure 5A**).

We then compared production of indolic compounds, including IAA, in *Pantoea* sp. YR343 and the Δ*crtB* mutant when grown in the presence of tryptophan. Wild type and mutant cells were grown to the same optical density and the supernatants were measured for the presence of IAA. Somewhat unexpectedly, we found that the Δ*crtB* mutant was defective in IAA production as determined using a colorimetric assay (**Figure 5B**). Indeed, we observed a nearly threefold decrease in indole production from 4.35 ± 0.20 μg/ml indoles in wild type cells compared to 1.52 ± 0.02 μg/ml indoles in Δ*crtB.*

**FIGURE 6:**
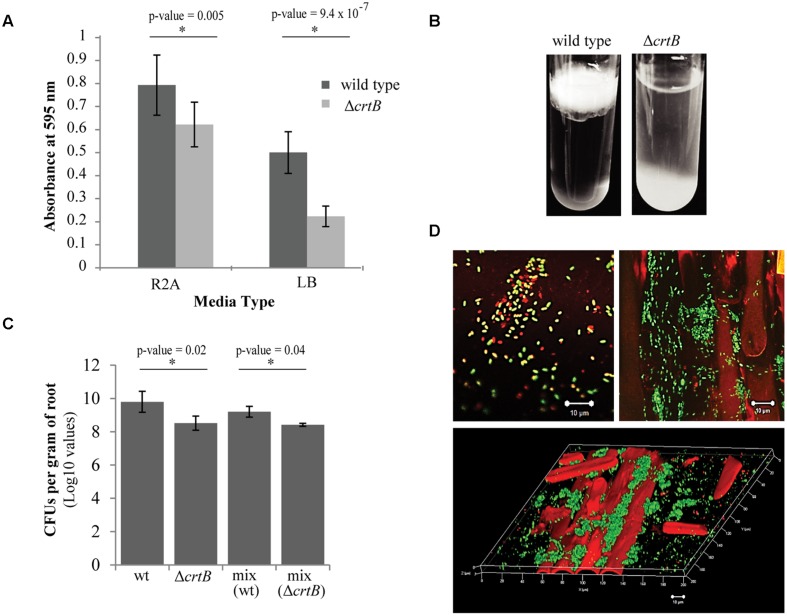
**Loss of carotenoids affects biofilm formation and root colonization. (A)** Comparison of biofilm formation between wild type *Pantoea* sp. YR343 and the Δ*crtB* mutant in plastic 96-well plates measured by the crystal violet assay **(B)** Pellicle formation assay performed in SOBG medium in glass test tubes. Wild type *Pantoea* sp. YR343 forms pellicles on top of the liquid medium, while the Δ*crtB* mutant settles to the bottom of the tube. **(C)** Wheat root colonization assay by wild type *Pantoea* sp. YR343 and Δ*crtB* is described as the Log10 value of colony forming units (CFUs) per gram of root material. **(D)** Images of wheat roots treated with YR343-GFP and Δ*crtB* for 1 week and stained with Syto61. Top left image shows a group of motile cells outside of the plant. Green represents the wild type population, while red represents the Δ*crtB* mutant population. Top right image shows a wheat root (visualized using autofluorescence in the red channel) colonized predominantly by wild typeYR343-GFP (green). Bottom image shows a three-dimensional view of a colonized wheat root.

We next examined the ability of wild type and Δ*crtB* cells to form biofilms using two different formats: biofilm formation on abiotic surfaces using 96-well plates with breathable tape, and biofilm formation at the air-liquid interface (pellicles) using glass test tubes. Using the 96-well plate assay, we found that the Δ*crtB* mutant was impaired in biofilm formation, with the defect more apparent when cells were grown in LB medium (**Figure 6A**). Likewise, Δ*crtB* mutant cells were also impaired in pellicle formation (**Figure 6B**). In this experiment, the mutant cells tended to settle at the bottom of the tube rather than form a biofilm at the air-liquid interface like wild type cells.

Due to the observation that the Δ*crtB* mutant is impaired in biofilm formation and IAA production, we speculated that this mutant may also be affected in root colonization. For these studies, wheat was used as the host plant due to its hardiness and ease of growth. Three plants were tested for each of four conditions, including uninoculated control, wild type only, Δ*crtB* only, and a 1:1 mix of wild type and Δ*crtB*. After 1 week, the roots were harvested and washed prior to plating in order to compare the amount of CFU per treatment. We found that the wild type cells colonized the roots significantly better than the Δ*crtB* mutant and this behavior is also observed in the 1:1 competition condition (**Figure 6C**).

Root colonization by *Pantoea* sp. YR343 wild type and Δ*crtB* mutants was also examined using microscopy. In this case, *Pantoea* sp. YR343 with a chromosomal insertion of EGFP was used as the wild type strain (YR343-GFP) and the plants were inoculated as described above. After 1 week, the roots were harvested, washed and stained with Syto61, which is a cell-permeable dye that fluoresces red. Cells that only fluoresced red were assumed to be Δ*crtB* mutants, and cells that fluoresced red and green were assumed to be wild type cells expressing GFP. Examination of the plant roots indicated that there were significantly more wild type cells (green) than mutant cells (red) attached to the roots (**Figure 6D**). These cells appeared to colonize in crevices on the plant root (**Figure 6D**) as shown in the 3D reconstruction from a z-stack using confocal microscopy (**Figure 6D**). Because of the predominance of wild type cells along the roots, we also examined the composition of cells in the wash based on the assumption that this might represent loosely attached cells. In this case, we found a mixture of wild type and Δ*crtB* mutant cells (**Figure 6D**), most of which appeared to be motile (data not shown). Similar results were obtained using two fluorescent strains, wild type YR343-GFP and the Δ*crtB* mutant strain with a chromosomal insertion of mCherry (data not shown).

## Discussion

*Pantoea* sp. YR343 was isolated from the rhizosphere of a healthy *Populus deltoides* tree in North Carolina and is a robust colonizer of plant roots. Based on phylogeny, *Pantoea* sp. YR343 is most closely related to *Pantoea* sp. GM01 which was isolated from the rhizosphere of a *P. deltoides* tree in Tennessee. Like *Pantoea* sp. YR343, the colonies of *Pantoea* sp. GM01 are yellowish in color and this strain is predicted to produce carotenoids based on its genomic sequence. Our phylogenetic analyses also indicated that both *Pantoea* sp. YR343 and GM01 are closely related to *P. rwandensis*, *P. rodasii*, *P. vagans*, and *P. eucalypti*, all of which were isolated from eucalyptus trees (Coutinho et al., 2002; Brady et al., 2009, 2012). Importantly, however, *P. rwandensis*, *P. rodasii*, *P. vagans*, and *P. eucalypti* were isolated from diseased eucalyptus trees and have been implicated as the causal agents of bacterial blight, leaf lesions, and dieback. Despite the close phylogenetic relationship, we have not observed any pathogenicity associated with *Pantoea* sp. YR343 using *P. deltoides*, *T. aestivum* (wheat), or *A. thaliana* as plant hosts. Since only two isolates are available from *Populus* in the Eastern USA at present, it is unclear at this point whether these isolates represent a single or multiple new species. It will be interesting to perform comparative genomic analyses on new and related *Pantoea* strains as they become available to resolve these systematics issues and to determine whether any host-specificity or pathogenicity-related factors can be identified.

*Pantoea* sp. YR343 possesses a number of characteristics that may promote its ability to survive in the rhizosphere and associate with plant hosts, including both swimming and swarming motility, the ability to solubilize phosphate, and the production of IAA. Motility, directed by chemotaxis, is an important means by which bacteria may avoid adverse conditions, while detecting and colonizing roots within the soil environment (Bashan, 1986; de Weert et al., 2002; Somers et al., 2004). Swarming motililty has been associated with virulence in the plant pathogen, *P. stewartii*, which colonizes the plant xylem, blocking flow and causing wilting in its plant hosts, including corn (Herrera et al., 2008). We observed enhanced swarming motility in *Pantoea* sp. YR343 in the presence of 0.4% glycerol, as compared to medium containing 0.4% glucose. Interestingly, this phenotype was the direct opposite of that shown for *P. stewartii* (Herrera et al., 2008), suggesting a possible difference in metabolism and preferred growth environment for *Pantoea* sp. YR343. *Pantoea* sp. YR343 can also solubilize phosphate, which has been implicated in plant-growth promotion due to increasing the availability of usable phosphate to the plants (Rodriguez and Fraga, 1999).

We also observed that *Pantoea* sp. YR343 produces cellulose during biofilm growth as a component of the exopolysaccharide matrix. Examination of *Enterobacteriaceae* genomes shows the existence of two distinct cellulose synthesis gene clusters, represented by the operon structure found in *Gluconacetobacter xylinus* (Wong et al., 1990) compared to the operon structure found in *Escherichia coli* (Zogaj et al., 2001). While the catalytic proteins, encoded by *bscA* and *bscB*, are conserved in both types of cellulose synthesis gene clusters, there are genes unique to each operon for which the functions are not well known (Romling, 2002). The majority of bacteria that produce cellulose possess a single cellulose synthesis gene cluster; however, *Pantoea* sp. YR343 possesses two gene clusters with distinct organizations representing both classes of cellulose synthesis gene clusters. Genomic comparisons indicate the presence of two cellulose synthase operons in other *Pantoea* spp. and some related *Klebsiella* spp. as well. Whether these cellulose gene clusters are differentially regulated and/or produce distinct cellulose synthase complexes has yet to be determined.

Our analyses indicate that *Pantoea* sp. YR343 readily forms biofilms on abiotic and biotic surfaces. Interestingly, our initial attempts at measuring biofilm formation using a 96-well assay were inconsistent until we replaced the lid of the 96-well plate with breathable tape. This observation suggests that oxygen-sensing may play a role in biofilm formation in *Pantoea* sp. YR343, although we have not yet explored this hypothesis in depth. Using microscopy and engineered strains of *Pantoea* sp. YR343 that express fluorescent proteins, we have observed the attachment of *Pantoea* sp. YR343 on plant root surfaces. On rare occasions, we have also observed *Pantoea* sp. YR343 within plants (data not shown), suggesting that it can survive as a plant endophyte, at least transiently.

The transient production of reactive oxygen species (oxidative bursting) is a common plant defense mechanism during the early stages of plant–microbe interactions (Doke et al., 1996; Lamb and Dixon, 1997; Wojtaszek, 1997; Torres et al., 2006; Nanda et al., 2010). Many beneficial microbes have developed a number of strategies for overcoming such plant defense mechanisms (Zamioudis and Pieterse, 2012). For example, oxidative stress has been overcome by up-regulation of antioxidant pathways in *Gluconaacetobacter diazotrophicus* (Alqueres et al., 2010), production of superoxide dismutase in *Rhizobium* species (Santos et al., 2000), and synthesis of alkyl hydroperoxide reductase in *Azospirillum brasilense* (Wasim et al., 2009). Similarly, many microbes produce carotenoids, which play a vital role in the survival of cells under harsh conditions, such as oxidative stress, extremes in pH, and resistance to toxins (Liaaen-Jensen and Andrewes, 1972; Krinsky, 1989; Kulkarni et al., 2015). Thus, we hypothesized that carotenoids may play a role in rhizosphere survival and/or plant association. Genomic analyses of *Pantoea* sp. YR343 identified candidate genes encoding all of the biosynthetic enzymes involved in carotenoid biosynthesis (To et al., 1994; Sedkova et al., 2005). As predicted from these analyses, loss of phytoene synthase activity by deletion of the *crtB* gene, resulted in a strain that was unable to produce any detectable carotenoids and showed increased sensitivity to reactive oxygen species.

The production of carotenoids by *Pantoea* sp. YR343 proved to be a distinguishing feature for Raman spectroscopic analysis, in that the signals produced from the presence of carotenoids, particularly zeaxanthin, dominated the spectra, as seen in **Figure 4D**. It is likely that the Raman intensities of the carotenoid bands are pre-resonantly enhanced, which is a Raman scattering process that occurs when the frequency of the excitation laser beam lies just below the frequency of an electronic transition of the chromophore in the irradiated molecule. This process results in the selective enhancement, by factors of up to 10^6^, of the Raman intensities of bands coupled to the electronic transition of the chromophore, which can be advantageous in the analysis of complex systems (Robert, 1990). Due to the selective enhancement afforded by resonance Raman scattering, molecular vibrations arising from other cellular components, such as DNA and proteins, are represented by relatively weak bands compared to the carotenoid bands in the *Pantoea* sp. YR343 Raman spectrum. The strong Raman signal in wild type *Pantoea* sp. YR343 also allowed us to follow the effect of H_2_O_2_ on carotenoids. In these analyses, we see a steady decrease in the Raman spectra associated with carotenoids as the cells are exposed to increasing concentrations of H_2_O_2_. The decrease in Raman signal intensity was apparent even at H_2_O_2_ concentrations that did not result in decreased cell viability, consistent with the protective role of carotenoids in the presence of free oxygen radicals (Krinsky, 1989, 1993; Hirayama et al., 1994; Edge et al., 1997).

Consistent with other *Pantoea* strains (Sergeeva et al., 2007; Kulkarni et al., 2013), we observed that *Panteoa* sp. YR343 produces IAA, which is synthesized from the amino acid tryptophan (Patten and Glick, 1996), an amino acid commonly found in plant root exudates (Kamilova et al., 2006). The genome of *Pantoea* sp. YR343 encodes a single, conserved *ipdC* gene which encodes indole pyruvate decarboxylase, suggesting that this strain synthesizes IAA using the indole pyruvate pathway (Patten and Glick, 1996). Other potential pathways to IAA production include the indole-acetamide pathway, although it is difficult to determine by genome comparisons whether this pathway is complete in *Pantoea* sp. YR343. Interestingly, IAA is produced in some pathogenic species of *Pantoea* that induce tumor or gall formation via the tryptamine pathway (Manulis et al., 1998). Our genome comparisons, however, suggest that *Pantoea* sp. YR343 does not have a complete tryptamine pathway. Surprisingly, we observed a decrease in IAA production by the Δ*crtB* mutant. This was unexpected since there was no obvious connection (e.g., common enzymes or substrates) between the pathways involved in carotenoid production and the pathways involved in IAA production. In addition to their protective role, however, carotenoids have been implicated in modulation of membrane fluidity and may play a role in the formation of membrane domains (Huang and Haug, 1974; Chamberlain et al., 1991; Gruszecki, 1999; Gruszecki and Strzalka, 2005; Landrum, 2009; Lopez and Kolter, 2010). From this perspective, the decrease in IAA production by the Δ*crtB* mutant may be a consequence of changes in membrane fluidity or organization. For example, uptake of tryptophan, which is a precursor to IAA production, may be defective in the Δ*crtB* mutant.

Likewise, we also observed changes in biofilm formation in the Δ*crtB* mutant. Indeed, the mutant was defective in both surface attachment and pellicle formation. In other organisms, such as *Bacillus subtilis*, it was shown that zaragozic acid, an inhibitor of squalene synthesis, could reduce the ability of *B. subtilis* to form biofilms (Lopez and Kolter, 2010). Squalene is a precursor in the production of hopanoids which, like carotenoids, have been show to modulate membrane fluidity and organization in bacteria (Kannenberg and Poralla, 1999; Kulkarni et al., 2015). Thus, we hypothesize that changes in membrane organization as a consequence of the loss of carotenoids could impact the function of membrane proteins involved in signaling and transport, and result in the observed defects in IAA production and biofilm formation in *Pantoea* sp. YR343.

Ultimately, the phenotypes associated with the loss of carotenoids in *Pantoea* sp. YR343 resulted in a mutant that was defective in plant association and/or rhizosphere survival. We observed a significant decrease in the number of Δ*crtB* mutant cells associated with plant roots compared to wild type based on both CFU counts and microscopy. There are several possible explanations for the role carotenoids may have in plant association. For example, carotenoids may be needed for survival of *Pantoea* sp. YR343 in the soil, which would therefore influence its ability to associate with a plant root. Alternatively, carotenoids may play a role in overcoming plant defense mechanisms, including oxidative bursting, during colonization of the root surface. That the ability to overcome oxidative stress is important for root colonization was shown in *Gluconoacetobacter diazotrophicus* PAL5 by demonstrating that production of superoxide dismutase and glutathione reductase were essential for colonization of rice roots (Alqueres et al., 2013). Another possibility is that the loss of carotenoids affects membrane organization and impacts the signaling pathways involved in plant recognition. Finally, the loss of carotenoids may affect the ability of *Pantoea* sp. YR343 to establish a strong interaction with the plant root, perhaps due to defects in IAA production or in biofilm production. Future studies are aimed at elucidating the mechanisms by which carotenoids impact membrane organization in *Pantoea* sp. YR343.

## Author Contributions

All authors contributed intellectual input and data analyses and assisted in manuscript preparation. JM-F, AB, and MD developed the experimental plan. AB, SF, DP, and JM-F performed phenotypic characterization and mutant analyses; CS and TC performed phylogenetic analyses, NE and TT performed GC-MS analyses, RM, SP, and PB performed Raman spectroscopy analyses, and AB, SJ, and DW conducted pathogenicity analyses. JM-F and AB wrote the paper.

## Conflict of Interest Statement

The authors declare that the research was conducted in the absence of any commercial or financial relationships that could be construed as a potential conflict of interest.
